# CIGB-300-Regulated Proteome Reveals Common and Tailored Response Patterns of AML Cells to CK2 Inhibition

**DOI:** 10.3389/fmolb.2022.834814

**Published:** 2022-03-11

**Authors:** Mauro Rosales, Arielis Rodríguez-Ulloa, George V. Pérez, Vladimir Besada, Thalia Soto, Yassel Ramos, Luis J. González, Katharina Zettl, Jacek R. Wiśniewski, Ke Yang, Yasser Perera, Silvio E. Perea

**Affiliations:** ^1^ Department of Animal and Human Biology, Faculty of Biology, University of Havana (UH), Havana, Cuba; ^2^ Molecular Oncology Group, Department of Pharmaceuticals, Biomedical Research Division, Center for Genetic Engineering and Biotechnology (CIGB), Havana, Cuba; ^3^ Mass Spectrometry Laboratory, Proteomics Group, Department of System Biology, Biomedical Research Division, CIGB, Havana, Cuba; ^4^ Biochemical Proteomics Group, Department of Proteomics and Signal Transduction, Max-Planck Institute of Biochemistry, Munich, Germany; ^5^ China-Cuba Biotechnology Joint Innovation Center (CCBJIC), Yongzhou Zhong Gu Biotechnology Co., Ltd., Yongzhou, China

**Keywords:** protein kinase CK2, kinase inhibitor, CIGB-300, acute myeloid leukemia, proteomics

## Abstract

Protein kinase CK2 is a highly pleiotropic and ubiquitously expressed Ser/Thr kinase with instrumental roles in normal and pathological states, including neoplastic phenotype in solid tumor and hematological malignancies. In line with previous reports, CK2 has been suggested as an attractive prognostic marker and molecular target in acute myeloid leukemia (AML), a blood malignant disorder that remains as an unmet medical need. Accordingly, this work investigates the complex landscape of molecular and cellular perturbations supporting the antileukemic effect exerted by CK2 inhibition in AML cells. To identify and functionally characterize the proteomic profile differentially modulated by the CK2 peptide-based inhibitor CIGB-300, we carried out LC-MS/MS and bioinformatic analysis in human cell lines representing two differentiation stages and major AML subtypes. Using this approach, 109 and 129 proteins were identified as significantly modulated in HL-60 and OCI-AML3 cells, respectively. In both proteomic profiles, proteins related to apoptotic cell death, cell cycle progression, and transcriptional/translational processes appeared represented, in agreement with previous results showing the impact of CIGB-300 in AML cell proliferation and viability. Of note, a group of proteins involved in intracellular redox homeostasis was specifically identified in HL-60 cell-regulated proteome, and flow cytometric analysis also confirmed a differential effect of CIGB-300 over reactive oxygen species (ROS) production in AML cells. Thus, oxidative stress might play a relevant role on CIGB-300-induced apoptosis in HL-60 but not in OCI-AML3 cells. Importantly, these findings provide first-hand insights concerning the CIGB-300 antileukemic effect and draw attention to the existence of both common and tailored response patterns triggered by CK2 inhibition in different AML backgrounds, a phenomenon of particular relevance with regard to the pharmacologic blockade of CK2 and personalized medicine.

## Introduction

Protein kinases are biological messengers that control multiple processes in cell physiology through reversible phosphorylation of thousands of proteins encoded by the human genome. In fact, phosphorylation is a pivotal mechanism for cell homeostasis, and its deregulation may result in aberrant signaling pathways implicated in a variety of human disorders (Cohen, 2002; Ardito et al., 2017).

Protein kinase CK2 (formerly known as casein kinase 2) is a ubiquitously expressed and constitutively active enzyme that exists as tetrameric complexes composed by two catalytic (α or αʹ) and two regulatory subunits (β) (Meggio and Pinna, 2003; Venerando et al., 2014). This highly pleiotropic Ser/Thr kinase is responsible for roughly 25% of cellular phosphoproteome, thus playing instrumental roles in normal and pathological states (Meggio and Pinna, 2003; Borgo et al., 2021). Of note, CK2 stands among the most studied kinases in recent years, and its contribution to the malignant phenotype and cancer progression has been suggested by mounting pieces of evidence (Trembley et al., 2009; Chua et al., 2017; Zonta et al., 2021). In particular, CK2 modulates signaling pathways critical for hematopoietic cell survival and function, and its high expression and activity in acute myeloid leukemia (AML) have been associated with worse prognosis and reduced overall survival (Kim et al., 2007; Quotti Tubi et al., 2017). Hence, in the past few years, CK2 has emerged as a promising candidate for molecular-targeted therapy in AML, a disease often characterized by poor long-term outcomes and resistance towards standard chemotherapy (Buontempo et al., 2018; Rosales et al., 2021a; Klink et al., 2021).

Importantly, the development of highly specific inhibitors has represented a major advance for CK2 substrate identification and elucidation of its roles in cell regulation (Gyenis et al., 2011). A number of CK2 inhibitors have been described so far, including small molecules targeting the ATP-binding site on the CK2α catalytic subunit (Sarno et al., 2011), several flavonoids characterized by a planar structure and hydroxylations at the 7 and 4′ positions (McCarty et al., 2020), and two synthetic peptides designed to antagonize the interaction between the CK2α and β subunits (Laudet et al., 2007) and bind the conserved acidic phosphoacceptor domain in CK2 substrates (Perea et al., 2018). The majority of such inhibitors have exhibited *in vitro* antiproliferative and proapoptotic activity, and some of them have also shown antitumor properties in animal models (Borgo and Ruzzene, 2021). In spite of the foregoing, only the ATP-competitive CK2 inhibitor CX-4945 and the synthetic peptide CIGB-300 have reached clinical trials in humans (Borgo and Ruzzene, 2021).

CIGB-300 is a peptidic inhibitor originally conceived to block the protein kinase CK2 activity through binding to the conserved acidic phosphoacceptor domain of substrates (Perea et al., 2004). The peptide is able to impair proliferation and viability of a variety of human cancer cells, including AML cells lines and primary cells from AML patients (Perea et al., 2008; Rosales et al., 2021a). However, pull-down assays and phosphoproteomic analysis have suggested that CIGB-300 is able to directly interact with the CK2α catalytic subunit and modulate the CK2-dependant phosphoproteome (Perera et al., 2020a; Perera et al., 2020b). Thus, CIGB-300 antineoplastic effect appears to be more complex than originally thought, owing to the convergence of both substrate binding mechanism and direct blockade of CK2 enzymatic activity. Considering the above, here we performed quantitative proteomic analysis in order to explore the molecular and cellular perturbations promoted by CK2 inhibition with CIGB-300 in two relevant AML backgrounds.

## Materials and Methods

### Cell Culture

Human leukemia cell lines HL-60 (American Type Culture Collection, Manassas, VA, United States) and OCI-AML3 (German Collection of Microorganisms and Cell Cultures, Braunschweig, Germany) were cultured in RPMI 1640 medium (Invitrogen, Carlsbad, CA, United States) supplemented with 10% (*v*/*v*) heat-inactivated fetal bovine serum (FBS) (Invitrogen, Carlsbad, CA, USA) and 50 μg/ml gentamicin (Sigma, St. Louis, MO, United States). Cells were maintained under standard cell culture conditions at 37°C and 5% CO_2_.

### Sample Preparation

For proteomic analysis, HL-60 and OCI-AML3 cells (10^7^ cells per each condition, three biological replicates) were incubated with 40 μM of the peptide CIGB-300 for 30 min and 3 h. Parallel to CIGB-300-treated groups, non-treated HL-60 and OCI-AML3 were incubated for 30 min and 3 h with vehicle and used as normalization control. Proteins from each replicate of CIGB-300-treated and non-treated AML cell groups were extracted with 1.5% SDS, 50 mM DTT, and boiling conditions for 10 min. Protein extracts were then processed by multienzyme digestion filter-aided sample preparation (MED-FASP) with overnight Lys-C and tryptic digestions (Wiśniewski and Mann, 2012). Protein and peptide concentrations were estimated by a tryptophan fluorescence-based assay previously described by Wiśniewski and Gaugaz (2015). Finally, 1 ng of peptides for each sample were injected for nanoLC-MS/MS analysis.

### NanoLC-MS/MS

A NanoLC EASY-nLC 1200 system coupled to a Q-exactive HF mass spectrometer (Thermo Scientific, Waltham, MA, USA) was used. Chromatographic runs for Lys-C and trypsin-derived peptides were performed in a home-made column (Dr. Maisch ReproSil-Pur C18-AQ 1.9 µm, 75 μm ID, 20 cm length) thermostated at 60°C. Peptides were eluted at 300 nl/min with a 120-min solution B (A: 0.1% formic acid in water and B: 0.1% formic acid in acetonitrile) gradient, starting at 5% solution B up to 30% in 95 min, then increased to 60% in 5 min, and finally up to 95% in 5 min more. A voltage of 2 kV was applied to the column tip to induce the nanospray and the mass range 300–1,650 *m*/*z* was scanned for data-dependent acquisition. Each mass spectrum obtained at 60,000 resolutions (20-ms injection time) was followed by 15 MS/MS spectra (28-ms injection time) at 15,000 resolutions. Proteins were only considered when detected in at least two replicates in any of the groups.

### Data Processing

Identification of peptides and proteins was based on the match-between-runs procedure using MaxQuant software (v1.6.2.10) (Cox et al., 2014), considering oxidation (M), deamidation (NQ), and N-terminal acetylation as variable modifications. Alignment of chromatographic runs was allowed with default parameters (20-min time window and a matching of 0.7 min between runs). Filtering and quantification were performed in Perseus computational platform (v1.6.2.2) (Tyanova et al., 2016). Student’s *t* test was employed to identify statistically significant changes (*p*-values lower than 0.05) in protein levels, after filtering for two valid values in at least one group. An additional cutoff of 1.5-fold change between CIGB-300-treated AML cells and the non-treated control was also applied.

### Bioinformatic Analysis

Differentially modulated proteins were tested for enrichment of Gene Ontology (GO Biological Processes) terms using Metascape gene annotation and analysis resource (https://metascape.org/), a web-based tool that computes the accumulative hypergeometric distribution and enrichment factors to identify significantly enriched biological processes through statistical analysis (*p*-value <0.01, enrichment factor >1.5) (Zhou et al., 2019). For either HL-60 or OCI-AML3 proteomic profiles, the Metascape Custom Analysis option was selected, and all identified proteins were used as background, and differentially modulated proteins at 30 min and 3 h after CIGB-300 treatment were combined and used as input dataset for meta-analysis. In addition, to represent the interaction networks associated with AML cell proteomic profiles, interactions among differentially modulated proteins were retrieved using Metascape, which compiles information from different integrative databases and applies the MCODE algorithm to extract highly connected regions embedded in each network (Bader and Hogue, 2003; Zhou et al., 2019). Finally, functional classification of CIGB-300-modulated proteins was based on the information retrieved through literature search and database curation, and protein interaction networks were visualized using Cytoscape software (v.3.5.0) (Shannon et al., 2003).

### Reactive Oxygen Species Detection

For detection of reactive oxygen species (ROS) levels in AML cells treated with the peptide, HL-60 and OCI-AML3 cells were incubated with 40 µM CIGB-300 during 30 min, 3 h, and 5 h. Following incubation, cells were collected by centrifugation, washed with PBS, and stained with the fluorescent probe dihydroethidium (DHE) (Sigma, MO, United States) for 30 min at 37°C in the dark. Finally, stained cells were analyzed in the Partec CyFlow Space flow cytometer (Sysmex Partec GmbH, Gorlitz, Germany), and FlowJo software (v7.6.1) (BD, Ashland, OR, USA) was used for data analysis and visualization. In all experiments, 5 mM H_2_O_2_ and 5 mM N-acetyl cysteine (NAC) anti-oxidant were used as controls.

### Annexin V/PI Staining

The viability of HL-60 cells treated with CIGB-300 in the presence or absence of NAC antioxidant was assessed using the FITC Annexin V Apoptosis Detection Kit I (BD Biosciences, San Jose, CA, United States). Briefly, cells were incubated with 40 µM CIGB-300 alone or in combination with 5 mM NAC for 30 min and 5 h. Cells were then washed twice with cold PBS and resuspended in binding buffer (1×) at 1 × 10^6^ cells/ml. Next, 5 µl of FITC Annexin V and 5 µl of propidium iodide (PI) were added, and cell suspensions were incubated for additional 15 min at room temperature in the dark. Analysis of stained cells and data processing/visualization were performed in the abovementioned Partec CyFlow Space flow cytometer and FlowJo software.

### Statistical Analysis

Differences between groups were determined using one-way ANOVA followed by Tukey’s multiple comparisons test. Analyses were performed in GraphPad Prism (v6.01) software for Windows (GraphPad Software, Inc, San Diego, CA, United States).

## Results

### Profiling CIGB-300-Regulated Proteome in AML Cells

To identify the array of proteins regulated by CIGB-300 in AML cells, we performed quantitative proteomic analysis of HL-60 and OCI-AML3 cells treated or not with 40 µM of this peptide inhibitor. A total of 6,270 and 6,181 proteins were identified in HL-60 after 30 min and 3 h of CIGB-300 treatment, respectively (Table 1; Supplementary Table S1). Likewise, 6,382 and 6,422 proteins were identified in OCI-AML3 cells at the same incubation periods (Table 1; Supplementary Table S1).

**TABLE 1:** Proteomic profile of AML cells treated with CIGB-300 peptide.

Proteome dataset	HL-60		OCI-AML3	
	30 min	3 h	30 min	3 h
Identified proteins	6,270	6,181	6,382	6,422
Significantly modulated proteins	25	85	74	58
	Total: 109; Overlap: 1		Total: 129; Overlap: 3	
Down-regulated	16	4	6	3
Up-regulated	9	81	68	55
—	Total: 233; overlap: 5			

Changes in protein levels between CIGB-300-treated and untreated cells were assessed using Student’s *t* test, and *p*-values below 0.05 were considered statistically significant. A fold-change threshold of 1.5 (|FC| ≥ 1.5) in treated vs control was also applied in order to define the down- and up-regulated proteins. As a result, in HL-60 cells, 25 and 85 proteins were identified as significantly modulated at 30 min and 3 h, while in OCI-AML3 cells, 74 and 58 proteins appeared differentially modulated in response to CIGB-300 treatment (Table 1; Supplementary Table S2).

**FIGURE 1:**
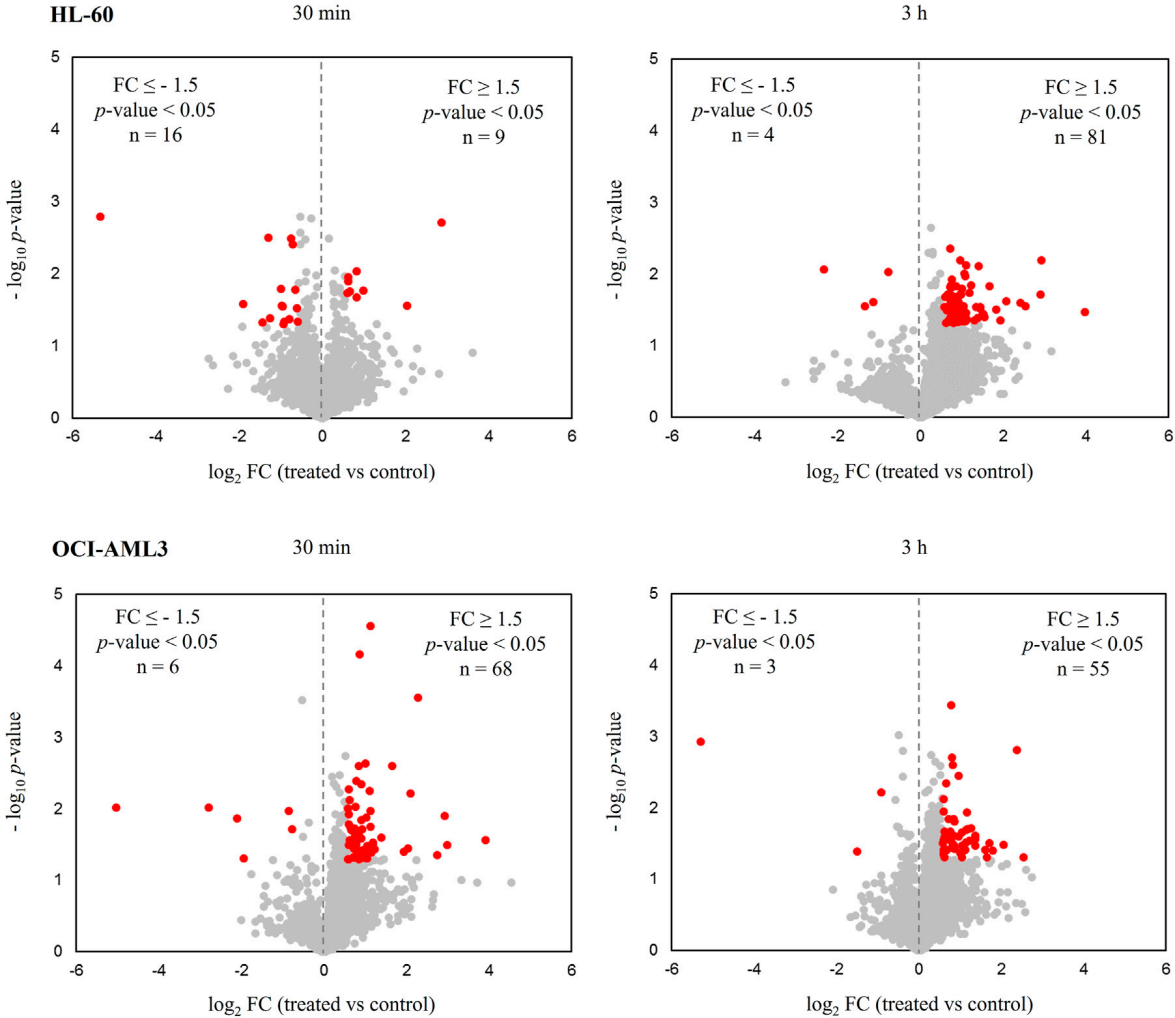
Proteomic profiles of human acute myeloid leukemia (AML) cells treated with the CK2 inhibitor CIGB-300. Volcano plots correspond to quantified proteins from HL-60 and OCI-AML3 cells after treatment with 40 µM of CIGB-300 for 30 min and 3 h. Red points indicate proteins that met the statistical significance cut-off (|FC| ≥ 1.5, *p*-value <0.05).

Of note, in practically all conditions, most of the differentially modulated proteins were up-regulated after CK2 inhibition with CIGB-300 as determined by distribution of down- and up-regulated proteins in volcano plots (Figure 1; Table 1). Exceptionally, in HL-60 cells treated with the peptide for 30 min, the number of down-regulated proteins was higher than the up-regulated ones (Figure 1; Table 1). Overall, a total of 109 and 129 proteins were differentially modulated in HL-60 and OCI-AML3 cells, respectively, with an overlap of one (MRPL52) and three (DCTPP1, GORASP2, and RAC1) proteins that appeared modulated at 30 min and 3 h after CIGB-300 treatment (Table 1; Supplementary Table S2). Besides, among all differentially modulated proteins, a total of five (CD53, HMGN1, NDUFC1, RCN2, and TPM3) overlapped between both cellular backgrounds (Table 1; Supplementary Table S2).

### Functional Characterization of CIGB-300-Regulated Proteome

**FIGURE 2:**
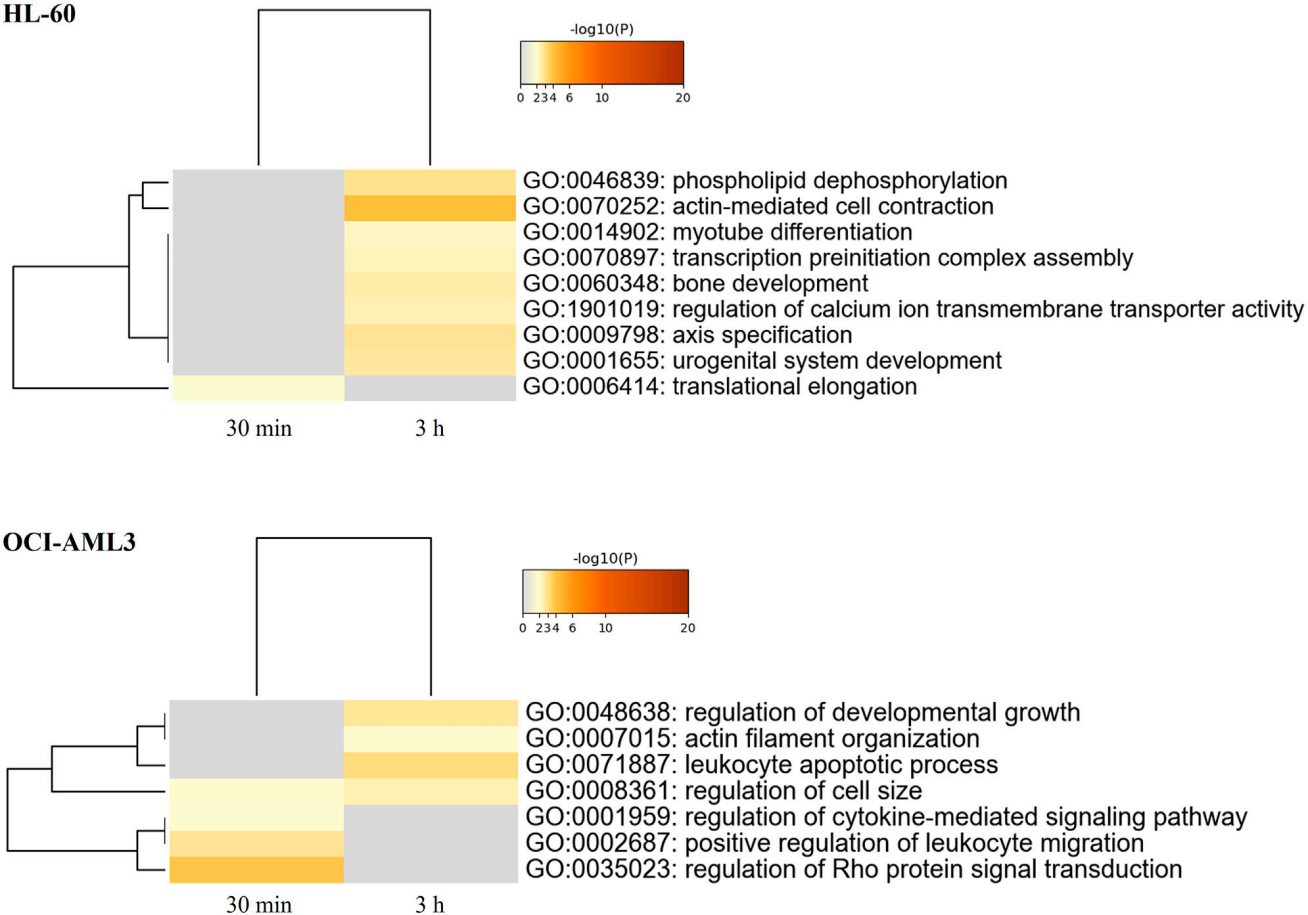
Heatmap of enriched terms (colored by *p*-values) across proteomic profiles of human AML cells treated with the CK2 inhibitor CIGB-300. Enrichment analysis for differentially modulated proteins in HL-60 and OCI-AML3 cells treated with 40 µM of CIGB-300 for 30 min and 3 h was based on annotations from Gene Ontology (GO) database. Biological processes significantly represented in proteomic profiles (*p*-value <0.01, enrichment factor >1.5) were identified using the Metascape gene annotation and analysis resource (https://metascape.org/).

Functional enrichment analysis of CIGB-300-regulated proteome in AML cells was performed using the Metascape bioinformatic tool (Zhou et al., 2019). In such analysis, actin-mediated cell contraction, phospholipid dephosphorylation, transcription preinitiation complex assembly, translational regulation, regulation of calcium ion transmembrane transport activity, and others were identified as biological processes significantly represented in HL-60 proteomic profile after CK2 inhibition with CIGB-300 (Figure 2; Supplementary Table S3). Besides, proteins involved in the regulation of Rho protein signal transduction, leukocyte migration, cytokine-mediated signaling pathway, developmental growth, and cell size, as well as leukocyte apoptotic process and actin filament organization, appeared differentially modulated in CIGB-300-treated OCI-AML3 cells (Figure 2; Supplementary Table S3).

**FIGURE 3:**
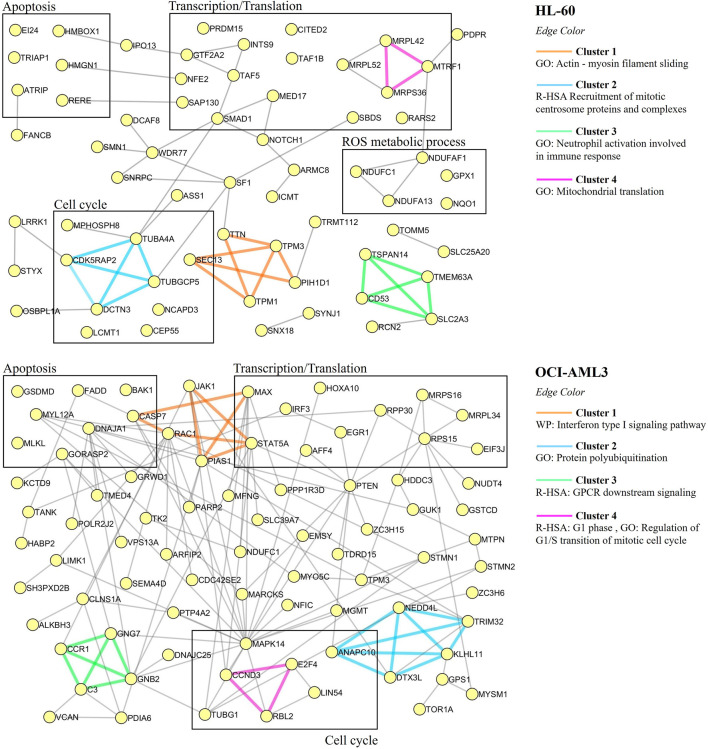
Protein–protein interaction networks associated with the proteomic profiles differentially modulated in AML cells in response to treatment with 40 µM of CIGB-300. Proteins are shown as yellow circles and clusters identified with the MCODE algorithm are highlighted with different edge colors. For each cluster, the related biological process or pathway according to annotations gathered from Gene Ontology (GO), Reactome (R-HSA), or WikiPathways (WP) databases are indicated.

To further characterize the proteomic profile regulated by CIGB-300, the interaction networks among differentially modulated proteins in HL-60 or OCI-AML3 cells were represented using Metascape tool, and highly connected regions in such networks were identified using the MCODE algorithm (Bader and Hogue, 2003; Zhou et al., 2019). As shown in Figure 3, clusters of proteins related to actin-myosin filament sliding (cluster 1), cell cycle (cluster 2), neutrophil activation (cluster 3), and mitochondrial translation (cluster 4) were differentially modulated in HL-60 cells after treatment with CIGB-300. Besides, the HL-60 proteomic profile also includes proteins related to apoptotic cell death, transcription, and ROS metabolic process (Figure 3). Similar results were obtained by functional enrichment analysis in which the biological processes of translation elongation, actin-mediated cell contraction, and transcription preinitiation complex assembly were found significantly represented in the proteomic profile of HL-60 cells (Figure 2; Supplementary Table S3).

On the other hand, network analysis of OCI-AML3 cell proteomic dataset evidenced that clusters of proteins related to interferon type I signaling (cluster 1), protein polyubiquitination (cluster 2), GPCR downstream signaling (cluster 3), and cell cycle (cluster 4) were differentially modulated in response to CK2 inhibition with CIGB-300 peptide (Figure 3). Furthermore, proteins related to apoptotic cell death were also identified in OCI-AML3-modulated proteome (Figure 3). In line with such result, cytokine-mediated signaling pathway and leukocyte apoptotic process were identified through functional enrichment analysis (Figure 2; Supplementary Table S3). Similar to HL-60 proteomic profile, besides proteins involved in cell cycle and apoptosis regulation, an array of proteins related to transcription and translation appeared modulated in CIGB-300-treated OCI-AML3 cells (Figure 3). Therefore, CK2 inhibition with CIGB-300 has an impact over these biological processes independently of the genetic background of these AML cell lines. Conversely, proteins involved in ROS metabolic process did not appear represented in OCI-AML3 proteomic profile.

### Differential Effect of CIGB-300 Over AML Cell ROS Production

**FIGURE 4:**
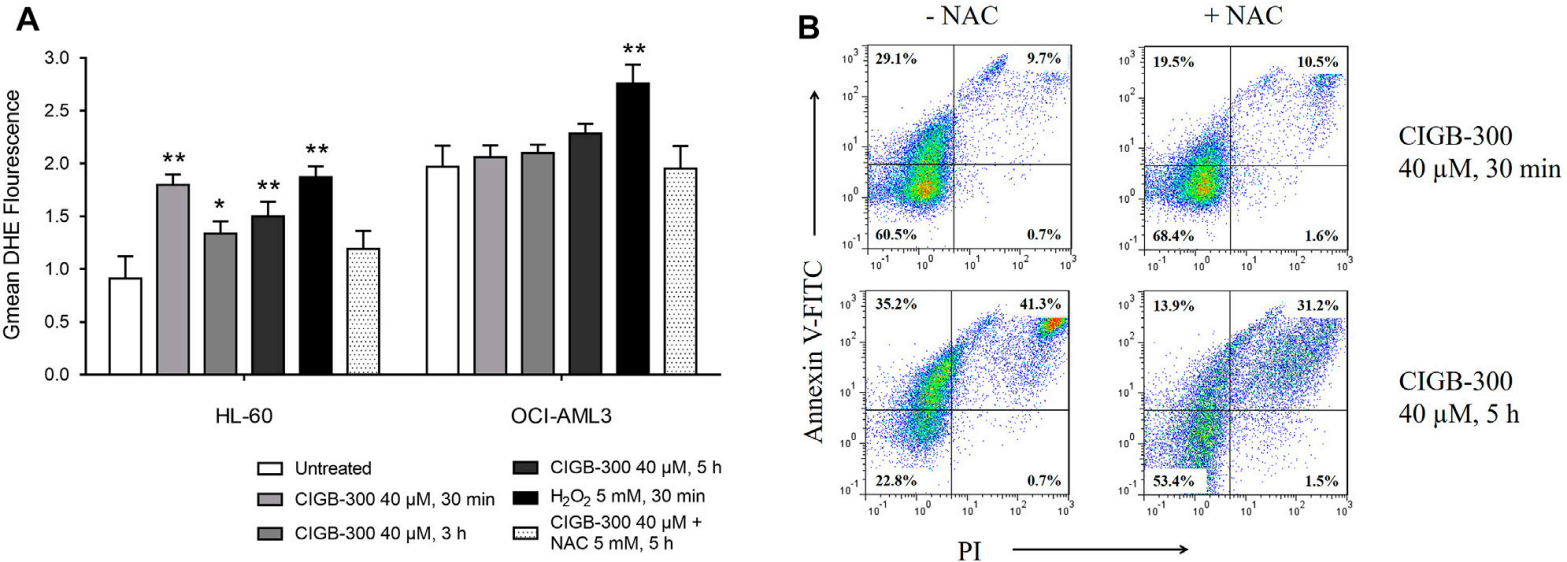
Evaluation of the impact of the CK2 inhibitor CIGB-300 over AML cell intracellular redox homeostasis. **(A)** Incubation with CIGB-300 peptide increased reactive oxygen species (ROS) production in HL-60 cells but not in OCI-AML3 cells. Intracellular ROS levels were determined by flow cytometry in AML cells incubated for the indicated times with CIGB-300 40 μM, H_2_O_2_ 5 mM, or CIGB-300 40 µM + NAC 5 mM. **(B)** The concomitant addition of CIGB-300 peptide and NAC anti-oxidant reduced the percentage of apoptotic HL-60 cells after 30 min and 5 h of treatment. Results from **(A)** are shown as mean ± SD, *n* = 3, and significant differences from comparison of each treatment with the corresponding untreated condition are indicated *p-value <0.05; **p-value <0.01.

Considering that proteomic analysis suggested a differential effect of CIGB-300 over ROS metabolic process in AML cells (Figure 3), we sought to elucidate if intracellular ROS accumulation could be related to CIGB-300 anti-leukemic effect. To determine ROS levels in HL-60 and OCI-AML3 cells after CK2 inhibition with CIGB-300, we use DHE as fluorescent probe for flow cytometry analysis. As a result, CIGB-300 treatment significantly increased ROS levels at all the assessed incubation times on HL-60 cells, with the highest intracellular ROS levels at 30 min of incubation (Figure 4A). Besides, H_2_O_2_ increased ROS levels and NAC anti-oxidant control abrogated ROS production in HL-60 cells treated with CIGB-300 (Figure 4A). On the contrary, in OCI-AML3 cells, CK2 inhibition with CIGB-300 did not induce any alteration of ROS homeostasis, which is in agreement with results from proteomic analysis (Figure 4A).

Of note, the potential connection between ROS de-regulation and CIGB-300-induced apoptosis was explored in HL-60 cells. Importantly, Annexin V/PI staining evidenced that the addition of NAC anti-oxidant reduced not only the accumulation of intracellular ROS but also the percentage of HL-60 cells undergoing apoptosis after 30 min and 5 h of treatment with the peptide (Figure 4B).

## Discussion

In the protein kinase landscape that has emerged as attractive targets for cancer treatment, CK2 stands among the most studies in recent years. This highly pleiotropic enzyme controls a number of signaling networks playing essential roles for malignant phenotype maintenance, and cancer cells often develop an excessive “addiction” to CK2 activity (Ruzzene and Pinna, 2010). The abovementioned has fostered the investigation of several CK2 inhibitors (Borgo and Ruzzene, 2021), including the clinical-grade synthetic peptide CIGB-300 (Perea et al., 2004). Accordingly, here we use quantitative proteomics to identify the CIGB-300-regulated proteome and explore the molecular perturbations promoted by this CK2 inhibitor in HL-60 and OCI-AML3 cells following 30 min and 3 h of treatment. These human cell lines derived from two relevant AML subtypes (i.e., acute promyelocytic and acute myelomonocytic leukemia), together accounting for roughly two thirds of all AML cases (Hanson et al., 2002). Of note, in proteomic profiles from both AML cell lines, CIGB-300 significantly modulated proteins related to apoptosis, cell cycle, transcription, and translation, while proteins involved in intracellular redox homeostasis were only identified in HL-60 cells.

Accumulated evidence demonstrates that CIGB-300 induces apoptosis in cancer cells, including AML cell lines and primary cells (Perea et al., 2004; Rosales et al., 2021a). In agreement with such results, mediators of intrinsic and extrinsic apoptotic pathways were both identified in the proteomic profile regulated by the peptide in AML cells. The pro-apoptotic proteins etoposide-induced 2.4 (EI24) and arginine-glutamic acid dipeptide repeats (RERE) were down-regulated at 30 min in HL-60 cells. EI24 is a p53 and E2F target gene that inhibits tumor progression through attenuation of NF-κB signaling (Choi et al., 2013; Sung et al., 2013), while RERE protein colocalizes with the pro-apoptotic protein BAX and triggers caspase-3 activation (Waerner et al., 2001). It is known that to counteract pro-apoptotic stimuli, cancer cells might activate pro-survival mechanisms, an event that could explain the decreased expression levels of EI24 and RERE proteins in response to CK2 inhibition with CIGB-300.

In line with these findings, TP53-regulated inhibitor of apoptosis 1 (TRIAP1) and ATR interacting protein (ATRIP) were up-regulated in HL-60 cells at 30 min and 3 h, respectively. TRIAP1 mediates the transport of phosphatidic acid across the intermembrane space for cardiolipin synthesis and inhibits the release of cytochrome-c from mitochondria during apoptosis (Potting et al., 2013). Furthermore, to inhibit apoptosis and allow DNA damage repair under low levels of genotoxic stress, TRIAP1 interacts with heat shock protein 70 (HSP70) and impairs the formation of the apoptosome complex (Park and Nakamura, 2005; Fook-Alves et al., 2016). On the other hand, ATRIP is an essential mediator of the DNA damage response, which inhibits replicative stress and TP53-dependent cell death (Matos-Rodrigues et al., 2020). As we mentioned before, proteomic data suggests that HL-60 cells treated with CIGB-300 seem to be subjected to increased levels of ROS, a known source of genotoxic stress, which could lead to apoptotic cell death (Srinivas et al., 2019). In such context, a pro-survival response could be based on the activation of DNA repair mechanisms to mitigate the accumulation of DNA damage.

In case of OCI-AML3 cell proteomic profile, the pro-apoptotic protein BCL2 antagonist/killer 1 (BAK) was up-regulated after 3 h. BAK1 promotes the formation of mitochondrial voltage-dependent anion channels leading to loss in membrane potential and release of cytochrome-c (Wei et al., 2001). Besides, as earlier as 30 min following CIGB-300 treatment, the expression of Fas-associated death domain protein (FADD) increased in OCI-AML3 cells. Such modulation of FADD protein abundance is of great relevance for CIGB-300 chemotherapeutic potentialities, since previous data indicate that absent or low FADD protein expression in leukemic cells is a prognostic factor for poor response of AML cells to chemotherapy (Tourneur et al., 2004). FADD activates pro-caspase-8 and the subsequent caspase signaling cascade in response to apoptotic signals initiated by activation of death receptors belonging to the family of tumor necrosis factor (TNF) receptors (Marín-Rubio et al., 2019). In addition to increased levels of BAK and FADD, the augmented abundance of caspase-7 and gasdermin-D (GSDMD) supports the pro-apoptotic effect of CIGB-300 in OCI-AML3 cells. Caspase-7 is an apoptotic executioner caspase, while the N-terminal fragment of GSDMD, which is cleaved by caspase-1, permeabilizes the mitochondrial membrane and leads to cytochrome-c release (Sborgi et al., 2016; Rogers et al., 2019).

Among proteins significantly regulated by the CK2 inhibitor CIGB-300, proteins related to cell cycle appeared represented in AML proteomic profiles. For instance, in HL-60 cells, differentially expressed proteins include a cluster (CDK5RAP2, DCTN3, TUBA4A, and TUBGCP5) involved in the recruitment of mitotic centrosome proteins and complexes. Other proteins related to transition through mitotic cell cycle like centrosomal protein of 55 kDa (CEP55) (Jeffery et al., 2016) and the heterochromatin component M-phase phosphoprotein 8 (MPHOSPH8) (Tchasovnikarova et al., 2015) were modulated in the presence of CIGB-300. The expression of leucine carboxyl methyltransferase one protein (LCMT1), which methylates the catalytic subunit of protein phosphatase 2A (PP2A) (Lee and Pallas, 2007), was increased after 3 h of CIGB-300 treatment. PP2A regulates G2/M transition of cell cycle and contributes to mitotic chromosome assembly by promoting chromosomal association of condensin-2 complex (Takemoto et al., 2009; Li et al., 2020). Of note, the condensin-2 regulatory subunit D3 (NCAPD3) was up-regulated at 3 h in HL-60 cells. Similarly, proteins differentially modulated in OCI-AML3 cells include a cluster (CCND3, E2F4, and RBL2) specifically involved in the G1/S transition. Cyclin D3 (CCND3), which functions as a regulatory subunit of CDK4 or CDK6, was up-regulated at 3 h after CIGB-300 treatment. Also, the transcription factor E2F4, a repressor member of the E2F family, its partner the retinoblastoma-like protein 2 (RBL2/p130), and the protein Lin-54 homolog (LIN54) were up-regulated in OCI-AML3 cells. Such proteins are subcomponents of the LIN complex (LINC), which represses G1/S and G2/M genes during G0 and early G1 phase of the cell cycle (Fischer and Müller, 2017).

The impact of CK2 inhibition over cell cycle in AML was previously corroborated using the ATP-competitive inhibitor CX-4945 and CIGB-300 peptide. In such studies, flow cytometry and phosphoproteomic analysis of AML cells evidenced an impairment of cell cycle progression in response to CK2 inhibition (Rosales et al., 2021a; Rosales et al., 2021b). In fact, the filament-forming cytoskeletal GTPase SEPTIN2 and the MCM2 subunit of the replicative helicase complex (MCM complex), both well-documented CK2 substrates related to cell cycle progression, were down-phosphorylated in CIGB-300-treated AML cells (Rosales et al., 2021a). Furthermore, as a downstream consequence of CK2 inhibition by CIGB-300, a significant number of down-phosphorylated phosphosites in AML cells were attributed to the CDK family, thus suggesting a functional impairment of such kinases (Rosales et al., 2021a).

Likewise, proteins related to transcription and translation were differentially modulated in both HL-60 and OCI-AML3 cells treated with CIGB-300. In agreement, previous results have evidenced an impact of CIGB-300 on such biological processes (Perera et al., 2015), and in AML cells, the peptide not only interacts with proteins from the small and the large ribosome subunits but also down-phosphorylates proteins required for transcription, ribosome biogenesis, and initiation of protein synthesis (Rosales et al., 2021a). Accordingly, several components of the transcription pre-initiation complex (TAF5, TAF1B, GTF2A, INTS9, and MED17) were modulated by CIGB-300 in HL-60 cells. Nuclear factor-erythroid 2 (NFE2), a transcription factor overexpressed in myeloproliferative neoplasms and important for hematopoietic stem cell maintenance and differentiation (Wang et al., 2010; Di Tullio et al., 2017; Peeken et al., 2018), was down-regulated in HL-60 cells. Furthermore, the transcriptional regulator Cbp/p300-interacting transactivator 2 (CITED2), which functions in stem cell maintenance and promotes leukemic cell survival (Korthuis et al., 2015; Mattes et al., 2017), was differentially modulated in HL-60 proteomic profile in response to CIGB-300 treatment. The modulation of such proteins suggests a potential role of CIGB-300 in regulating the self-renewal of leukemic stem cells.

In line with this hypothesis, the expression of the transmembrane receptor Notch1 was increased in HL-60, and tetraspanin 14 (TSPAN14) and manic fringe glycosyltransferase (MFNG), which function as positive regulators of Notch signaling pathway (Taylor et al., 2014; Jouannet et al., 2016), were up-regulated by CIGB-300 treatment in HL-60 and OCI-AML3 cells, respectively. The classical view sustains that Notch signaling keeps cells in an undifferentiated state and plays oncogenic roles in many cancers, including hematological malignancies (Ntziachristos et al., 2014). However, specifically in AML, accumulating pieces of evidence suggest that Notch signaling pathway has a tumor suppressor role (Kannan et al., 2013; Lobry et al., 2013). In such respect, Notch1 is down-regulated in AML cell lines and patient samples and impairs leukemogenesis of AML by increasing the expression of the transcriptional factor PU.1, which mediates myeloid differentiation (Yang et al., 1992; Tohda and Nara, 2001). Since leukemic stem cell maintenance has been associated with AML relapse and resistant phenotypes to chemotherapy, a modulation of Notch signaling pathway by CIGB-300 could add a benefit to the standard antileukemic therapies.

Similar to HL-60 proteomic profile, CIGB-300 modulates the abundance levels of several transcription factors in OCI-AML3 cells including the Myc interacting factor X (MAX) and the AF4/FMR2 family member 4 (AFF4) (Lin et al., 2010; Diolaiti et al., 2015). In addition, the transcription factor homeobox A10 (HOXA10), a protein related to leukemogenesis and chemoresistance that has been proposed as a prognostic marker for AML patients (Yi et al., 2016; Guo et al., 2020), was also differentially modulated. Of note, the early growth response protein 1 (EGR1), which functions as a tumor suppressor in AML (Gibbs et al., 2008; Tian et al., 2016), was up-regulated in OCI-AML3 cells in response CIGB-300. EGR1 promotes myeloid differentiation and suppresses the leukemic phenotype driven by the oncogenes c-Myc or E2F-1 (Gibbs et al., 2008). This transcription factor is known to activate the expression of other tumor suppressor genes including p53 and the phosphatase and tensin homolog (PTEN), promoting growth arrest or cell death in cancer cells (Wang et al., 2021). Interestingly, the abundance of PTEN, which is an essential tumor suppressor in human myeloid malignancies (Morotti et al., 2015), was increased in OCI-AML3 cells. Noteworthy, sustained activation of AKT by PTEN deficiency mediates the chemoresistance of AML cells to idarubicin and cytarabine anticancer drugs (Ryu et al., 2019). Therefore, the up-regulation of PTEN in response to CIGB-300 treatment supports the benefit of combining CIGB-300 with standard chemotherapy drugs.

Remarkably, an array of proteins related to oxidative phosphorylation was only identified in HL-60 cells. Among these proteins, two subunits of the NADH:ubiquinone oxidoreductase/complex I (NDUFA13 and NDUFC1) were up-regulated in response to CIGB-300. The complex I catalyzes the electron transfer from NADH to ubiquinone in the first step of the mitochondrial respiratory chain, and its subunit NDUFA13, in addition to be required for electron transfer (Lu and Cao, 2008), functions as a tumor suppressor that binds to STAT3 and inhibits its transcriptional activity (Nallar et al., 2010). Together with NDUFA13 and NDUFC1 subunits, complex I intermediate-associated protein 30 (NDUFAF1), a mitochondrial chaperone involved in the assembly and stability of complex I (Vogel et al., 2005), was also up-regulated. The concomitant up-regulation of these proteins suggests that complex I could have an increased activity in CIGB-300-treated HL-60 cells, which could lead to an over-production of ROS since complex I is one of the main sources of mitochondrial oxidative stress (Brand, 2016). Importantly, such finding was corroborated by flow cytometry using the fluorescent probe DHE to determine ROS levels in AML cells.

Along with increased ROS levels, the cellular antioxidant defenses can be activated as a compensatory mechanism. In line with this, glutathione peroxidase (GPX1) and NAD(P)H dehydrogenase [quinone] 1 (NQO1) were up-regulated in HL-60 cells. The enzyme GPX1 detoxifies the hydrogen peroxide (H_2_O_2_) generated by superoxide dismutase (Arthur, 2001), while NQO1 reduces quinones to hydroquinones, preventing the generation of radical oxygen species (Ross and Siegel, 2021). Notably, AML cells have a reduced spare respiratory capacity in comparison with normal hematopoietic cells, and increasing electron flux through the respiratory chain preferentially promotes oxidative stress and induces cell death (Sriskanthadevan et al., 2015). Accordingly, we demonstrated that the reduction of intracellular ROS production by NAC anti-oxidant was accompanied by a reduced percentage of HL-60 cells undergoing apoptosis following treatment with CIGB-300. Thus, the modulation of proteins related to oxidative phosphorylation could mediate the antileukemic effect of CIGB-300 and promote apoptosis in HL-60 cells. Nevertheless, the addition of the anti-oxidant does not completely abrogate the pro-apoptotic effect of CIGB-300, evidencing that other molecular mechanisms different from intracellular ROS production could also be responsible for the induction of apoptosis in HL-60 cells. The foregoing is confirmed by the induction of apoptosis observed in OCI-AML3 cells (Rosales et al., 2021a), in spite of the absence of ROS accumulation when treated with CIGB-300.

Altogether, our proteomic analysis supports previous results evidencing that the proapoptotic effect and the impact of CIGB-300 over cell cycle regulation and transcriptional/translational processes are a common denominator for CK2 inhibition in AML cells (Rosales et al., 2021a). Conversely, modulation of proteins involved in redox homeostasis was only observed in HL-60 cells. Such findings not only provide fresh clues related to CIGB-300 antileukemic effect but also highlight that CK2 inhibition with the CIGB-300 triggered common and tailored response patterns in different AML backgrounds.

## Data Availability

The original contributions presented in the study are included in the article/Supplementary File, further inquiries can be directed to the corresponding authors.
